# Differentiation Between Tendinous, Myotendinous and Myofascial Injuries by L-BIA in Professional Football Players

**DOI:** 10.3389/fphys.2020.574124

**Published:** 2020-09-04

**Authors:** Lexa Nescolarde, Joaquim Terricabras, Sandra Mechó, Gil Rodas, Javier Yanguas

**Affiliations:** ^1^Department of Electronic Engineering, Universitat Politècnica de Catalunya, Barcelona, Spain; ^2^Medical Department, Futbol Club Barcelona, FIFA Medical Center of Excelence, Barcelona, Spain; ^3^Department of Surgery, Faculty of Medicine, Universitat Autònoma de Barcelona, Barcelona, Spain; ^4^Department of Radiology, SCIAS–Hospital de Barcelona, Barcelona, Spain

**Keywords:** localized bioimpedance, MRI, myotendinous junction, grade of muscle injury, professional football players

## Abstract

**Purpose:**

To differentiate by localized bioimpedance (L-BIA) measurements 24 h after injury, between tendinous, myotendinous junction (MTJ), and myofascial junction (MFJ) injuries, previously diagnosed by MRI exam. To evaluate by L-BIA, the severity of MTJ injuries graded from 1 to 3, and to determine the relationship between days to return to play (RTP) and L-BIA measurements.

**Methods:**

3T MRI and tetra polar L-BIA was used to analyzed 37 muscle injuries 24 h after injury in 32 male professional football players, (23.5 ± 1.5 kg m^–2^; 1.8 ± 0.1 m; 20–30 year.) between the 2016–2017 and 2017–2018 seasons. Muscle injuries were classified by *The British Athletics Muscle Injury Classification* (BAMIC). Percentage difference of L-BIA parameters [resistance (R), reactance (Xc), and phase angle (PA)] of the injured side were calculated considering contralateral non-injured side as the reference value.

**Results:**

According to BAMIC classification and by MRI exam, we found tendinous (*n* = 4), MTJ (*n* = 26), and MFJ (*n* = 7) muscle injuries. In addition, MTJ injuries were grouped according to the severity of injury in grade 1 (*n* = 11), grade 2 (*n* = 8), and grade 3 (*n* = 7). Significant decrease (*P* < 0.01) was found in the L-BIA parameters R, Xc, and PA, in both MTJ and MFJ as well as in the different grades of MTJ injuries. In particular, in Xc (*P* < 0.001), which is related to muscle cell disruption. Regarding days to RTP, there was statistical significance among the three different grades of MTJ injuries (*P* < 0.001), especially when grade 1 was compared to grade 3 and grade 2 compared to 3.

**Conclusion:**

L-BIA is a complementary method to imaging diagnostic techniques, such as MRI and US, to quantify MTJ and MFJ injuries. In addition, the increase in the severity of the MTJ injury resulted in higher changes of the Xc parameter and longer time to RTP.

## Introduction

Muscle injures of lower limbs are the most common injury types in athletes (Junge et al., 2009; Engebretsen et al., 2013), accounting for more than 30% of injuries in professional football players (Ekstrand et al., 2011; Elliott et al., 2011). This trend seems to keep growing in professional male football players in Europe (Ekstrand et al., 2016).

An optimal diagnosis of muscle injury determines the severity of the injury, time to return to play (RTP) and risk of re-injury. However, determining the optimal RTP in professional football players is difficult due to the variability and complexity of the process. The decision of whether an athlete can safely RTP remains challenging (Askling et al., 2010) with ∼ 60% of re-injuries few weeks after RTP (Brooks et al., 2006; Heiderscheit et al., 2010). Imaging, either through magnetic resonance imaging (MRI) and/or ultrasound imaging (US) is key to assess muscle injuries and prognosis. However, it is still debated whether MRI report is a good predictor for time to RTP (Reurink et al., 2014). This is probably due to the lack of consensus in the diagnosis and classification of muscle injuries.

In the last 8 years, there have been proposed multiple different systems of muscle injury classifications based on clinical examination and radiological findings, especially through MRI (Mueller-Wohlfahrt et al., 2013; Pollock et al., 2014; Valle et al., 2017). The *British Classification of Muscle Injuries of Athletics, BAMIC* (Pollock et al., 2014), based on MRI findings, is one of the most internationally used and classifies muscle injuries according to anatomical location: myofascial, muscle-tendon junction, and intratendinous; as well as according to the severity of the injury, grading from 0 to 4. Moreover, the BAMIC specifies that each hamstring muscle injury localization has different RTP. The criteria is based on the tissue type involved and the extent of the injury, being the intratendinous injury the one with longer time to RTP and higher recurrence rate (Patel et al., 2015; Pollock et al., 2016; Wangensteen et al., 2017).

The classification proposed by Pollock et al. (2014) agree with the idea of a histoarchitectural approach to skeletal muscle injury from Balius et al. (2020) describing that when myoconnective junction is affected, the injury can be located either in a MTJ (the injury affects an aponeurosis or a tendinous expansion attached to muscle fibers) or in the myofascial junction (MFJ) (when the injury involves muscle fiber and their perimysium and/or epimysium). And when an injury in the myoconnective junction has a tendon gap (by MRI), the injury will have worst prognosis, require longer time to RTP and could present greater risk of re-injury compared to others injuries. However, a study of full-thickness intramuscular tendon damage, did not show re-injury after 12 months (Vermeulen et al., 2019). Regarding MTJ and MFJ injuries, it is not clear which one has better prognosis and, consequently, less time to RTP (Balius et al., 2020).

Mono-frequency non-invasive electrical bioimpedance is associated with the injection of low-intensity sinusoidal current at 50 kHz into a biological conductor resulting in measuring values of resistance (R), associated with the hydration state of soft tissue, and reactance (Xc) associated with the integrity of soft tissue structures (Lukaski, 1996; Lukaski and Piccoli, 2012, Lukaski, 2013). The current is delayed by the current flowing through the cell membranes (capacitive element, C). The $X_{\text{c}}=\frac{1}{2\,\pi \,f\,C}$is an indirect measure of the amount of applied current that penetrates to the cell membranes (Lukaski et al., 2019). That is, bioimpedance is a complex number *Z* = R−jXc, whose module, *Z* is obtained by $\sqrt{{\left(R\right)}^{2}+{\left(X\,c\right)}^{2}}$ and phase angle (PA) is the tan−1XcR.

When bioimpedance measurements are taken on a specific human body area, they are known as localized bioimpedance measurements (L-BIA). One of the first clinical applications of non-invasive L-BIA at 50 kHz is related to electrical impedance myography (EIM) in neuromuscular diseases demonstrated significantly reduced values of R, Xc, and PA due to muscle fiber atrophy, connective tissue accretion, fat infiltration, and edema (Rutkove et al., 2008; Rutkove, 2009). Lukaski and Moore (2012), using L-BIA with phase-sensitive BIA analyzer at 50 kHz, identified in lower leg wounds, a moderate decrease in R, Xc, and PA acutely after removing damaged tissue from a wound. Notably, the decreased occurred during infection and R, Xc, and PA values were restored after healing. Therefore, L-BIA could provide a safe and effective method to detect changes at cellular level.

In professional football players (Nescolarde et al., 2013, 2015), L-BIA measurements using 50 kHz phase-sensitive BIA analyzer, are in agreement with the severity of muscle injury, diagnosed by MRI 24 h after injury, according to muscle gap and independently to anatomical location. L-BIA measurements 24 h after injury showed a significant reduction of R, Xc and PA directly related to the severity of muscle injuries, being more noticeable in Xc than in R. Moreover, more severe injuries showed larger percentage difference in Xc (% difference) (Nescolarde et al., 2013, 2015). Sometimes there is a great challenge for the sports physicians and radiologists to be able to confirm diagnosis and especially the prognosis. Accurately quantifying the severity of muscle injury could help to improve the diagnosis of muscle injury.

This work aims (1) to differentiate by L-BIA, according to anatomical location, between tendinous, MTJ and MFJ injuries, previously diagnosed by MRI exam 24 h after injury; (2) to determine the severity of MTJ injuries graded from 1 to 3 by L-BIA; and (3) to determine the relationship between days to return to play (RTP) and L-BIA measurements 24 h after injury with severity of the injury.

## Materials and Methods

### Participants

Futbol Club Barcelona (FCB) Medical Department studied prospectively 37 muscle injuries of 32 male professional football players, (23.5 ± 1.5 kg m^–2^; 1.8 ± 0.1 m; 20–30 year.) between the 2016–2017 and 2017–2018 seasons to assess and compare L-BIA and MRI findings. This study only evaluates injuries of football players (both during matches and training sessions) that presented acute muscle injury, to whom the medical team prescribed an MRI in the first 24 h after the injury, and in those cases that L-BIA study was performed during the same period of time.

### Magnetic Resonance Imaging (MRI)

Magnetic resonance imaging is not only the gold standard technique of choice in professional football for diagnosing muscle injuries that has proven to be an essential tool in the assessment of muscle injuries in elite athletes, but is also rapidly becoming the imaging technique of choice for the evaluation of the connective tissue injuries (Schneider-Kolsky et al., 2006; Ekstrand et al., 2012). This imaging technique allows specialists to define muscle injury with excellent resolution of the three axes, including oblique planes, and assessing deep muscles by identifying the injury site from the origin, proximal myotendinous junction, muscle belly, distal junction or insertion point (Ahmad et al., 2013).

#### MRI Protocol

Magnetic resonance imaging was performed on 3T Canon Vantage Titan (Canon Medical Systems, Japan) in FCB Medical Center by a specialist musculoskeletal radiologist (SM) to classify the muscle injury. The specific MRI paramentrs are a maximum gradient strength of 45, 203 T/m/s Slew Rate, and 32 receiver channels. Axial, Sagittal and Coronal T2 Fat Sat, TR 5200, 5000, and 3700 ms, TE 44–60 ms, Eco train 7.5, SL 2.5–3.5 mm, in-plane resolution 0.9–1.4 mm× 0.88–0.97 mm, FOV 256 × 256, 192 × 272, 288 × 320 mm, and Axial and Coronal TSE T1, TR 900–980 ms, TE 11 ms, Eco train 7.5, SL 2.5–3.5 mm, in-plane resolution 0.71–0.9 mm × 0.71–0.9 mm, FOV 352 × 352, 288 × 320 mm was acquired. On T2 Fat Sat sequences, muscle injuries are detected by the presence of hyperintense fluid accumulation. It is recommended to perform fluid-sensitive sequences (T2-weighted in our case) with intermediate echo time (TE) (e.g., less than 65 ms) to obtain an adequate contrast and spatial resolution of connective tissues. With these sequences we are able to detect edematous changes around the myotendinous, myoaponeurotic, and myofascial junctions, identify small tears in the connective tissue, as well as to delineate intra- or inter-muscular fluid collections or hematomas. T1-weighted sequences are useful in the assessment of hematic collections, in the detection of fatty muscular infiltration, as well as in the detection of scar tissue in chronic injuries that could be detected as chemical shift artifacts in the T2-weighted sequences.

### Muscle Injury Grouping Through MRI Exam 24 h After Injury

After MRI exam, 37 muscle injuries were categorized according to *The British Athletics Muscle Injury Classification* (Pollock et al., 2014) and considering the histoarchitectural approach to skeletal muscle injury (Balius et al., 2020) taking to account:

1. Myoconnective junction affected

• Tendinosis injuries are tears that extend into the tendon.
• Myotendinous junction (MTJ) injuries are high signal change occurs within the muscle or more commonly located exactly at the MTJ.
• Myofascial junction (MFJ) injuries extend from the fascia and demonstrate high signal change on fat-suppressed/STIR sequences within the periphery of the muscle.

2. Severity of the injury, graded from 1 to 4

• Grade 1 injuries are small tears. High signal in MRI extends less than 10% of the cross-sectional area (CSA) of the muscle and over a limited area of less than 5 cm.
• Grade 2 shows hyper signal in MRI measure between 10 and 50% of CSA or extends between 5 and 15 cm within the muscle with a fiber disruption (gap) less than 5 cm.
• Grade 3 injuries show high signal change patterns greater than 50% of the CSA or greater than 15 cm in length.
• Grade 4, complete muscle or tendon tears are not included in this study.

### Localized Bioimpedance Measurements (L-BIA)

Tetra polar L-BIA measurements, both in the injured side and the non-injured side, were performed 24 h after occurrence of the injury to quantify the percentage difference in R, Xc, and PA of injured side respect to the contralateral non-injured side. The impact of the severity of muscle injury was characterized by the percentage difference in L-BIA (R, Xc, and PA). All injuries were diagnosed 24 h after injury by MRI exam. The variability of R and Xc (Nescolarde et al., 2015) between left and right non-injured muscle area were studied in 10 non-injured football players on three different days with an interval of 7-day between each measurement. The intra-individual differences in 1/3 proximal quadriceps, 1/3 medium quadriceps, 1/3 proximal hamstrings and 1/3 medium hamstrings ranged from −2.1 to 0.5 Ω and the inter-individual differences were lower than 15% (2 SD).

The measurements were taken with a phase-sensitive impedance instrument BIA 101 Anniversary (Akern-Srl, Florence, Italy) that applied a constant sinusoidal alternating current of 245 μARMS at 50 kHz. The range of measure for resistance is 0–1500 Ω and for reactance 0–500 Ω, with 2% maximum tolerance. Measurement errors determined with a parallel circuit of precision resistor and capacitor, were <1 Ω for R and <2% for Xc.

**FIGURE 1:**
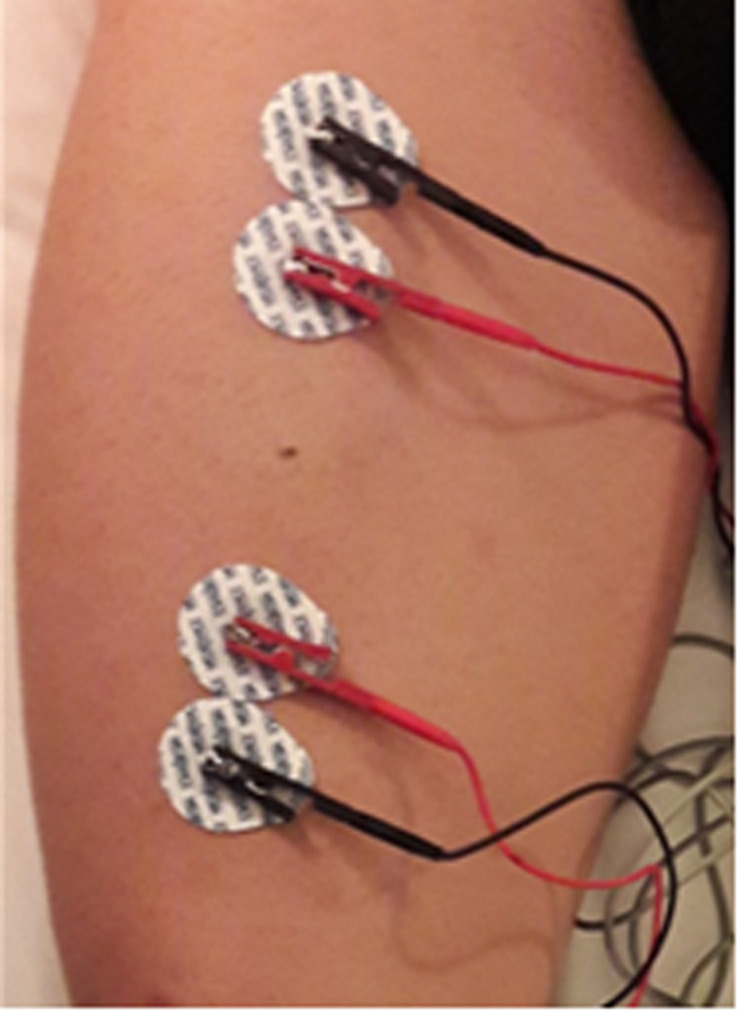
Tetra-polar L-BIA electrode placement for intramuscular septum rectus femoris (MTJ).

The adhesive contact electrode Ag/AgCl (COVIDIEN Ref. 31050522, COVIDIEN llc, Mansfield, IL, United States) with R and Xc intrinsic values of 10.89 and 0.30 Ω respectively (Nescolarde et al., 2016), was chosen for L-BIA measurements. Similar to previous reports (Nescolarde et al., 2015, 2017) detector voltage electrodes (red) were placed 5 cm proximally and 5 cm distally, respectively, from the center of the injury located by US Aplio i800 (Canon Medical Systems, Japan). Two current injecting electrodes (black) were placed close to the detector voltage electrodes (Figure 1). Because of the proximity of the current-introducing and voltage-detecting electrodes, the inter-tester reproducibility (Nescolarde et al., 2013) of the R and Xc measurements was evaluated by 5 examiners. The range of variability was negligible, 1 Ω for R and 0.45 Ω for Xc. The mean ± SD value of R and Xc were 39.6 ± 0.6 and 14.2 ± 0.5 Ω respectively, with coefficients of variation of 1.4% for R and 3.2% for Xc.

In injuries located near the bone detector, voltage electrodes were placed 10 cm proximally and 10 cm distally, respectively, from the center of the injury to increase the sensitivity of the measurement (Sanchez et al., 2016; Rutkove et al., 2017).

### Injury Diagnosis and Return to Play Criteria

Injuries were diagnosed by the medical team of the FCB Medical Department based on clinical history, physical examination, and US and MRI assessment. The establishment of return to play (RTP) criteria was addressed by the FCB Medical Department and team physicians follow the criteria expressed in FCB Muscle Guide 2019 (Barça Innovation Hub, 2020). The RTP (in days) started when the injury occurred and continued beyond the point when the player made his return to unrestricted match play (Hägglund et al., 2005). The RTP plan of FCB is individual avoiding re-injuries due to premature reinstatements.

### Data Analysis

The normality of distribution in the variables was determined by the Shapiro-Wilk test and the homogeneity of variances by Levene’s test. Normally distributed variables are shown as mean ± SD.

Repeated measures ANOVA test 2 × 3 was used to determine statistical differences between injured side respect to non-injured side in R, Xc, and PA considering contralateral non-injured side as reference value. In addition, different grades of severity (grades 1 to 3) of MTJ injuries were analyzed following the same statistical procedure.

One-way ANOVA test was used to determine statistical differences in the percentage difference of R, Xc, PA, and RTP values, between tendinous, MTJ, and MFJ injuries, as well as between different grades of severity of MTJ injuries.

Both, repeated measures ANOVA and One-way ANOVA test, with multiple comparison tests by Bonferroni (homogeneity variances assumed) or Tamhane’s T2 test (homogeneity variances not assumed) were used.

Discriminant Function Analysis was used to find a linear combination of features that separates muscle injuries L-BIA values (% difference in R, Xc, and PA) according to the grade of severity of MTJ injuries.

The statistical software IBM^®^ SPSS^®^ version 22.0 (IBM Corp, Armonk, NY, United States) was used for data analysis. The level of statistical significance was set at *P* < 0.05.

## Results

### Tendinous, MTJ and MFJ Injuries, Previously Diagnosed With MRI 24 h After Injury

**FIGURE 2:**
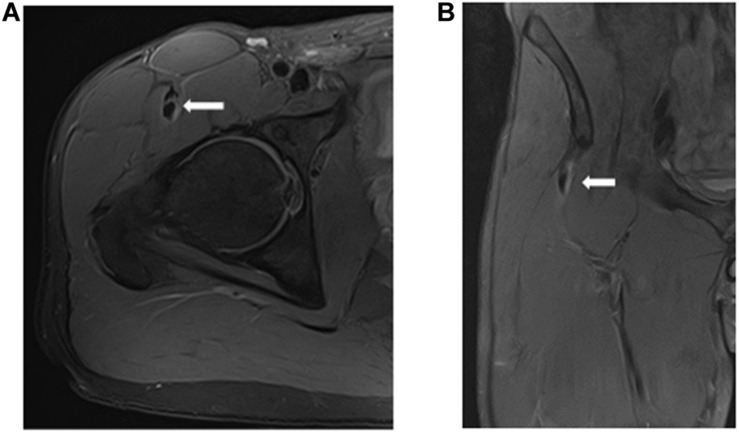
Tendinous injury of the central tendon of rectus femoris (longitudinal tear of the central tendon with slight peritendinous edema) in axial **(A)** and coronal **(B)** T2 weighted fat-suppressed images.

**FIGURE 3:**
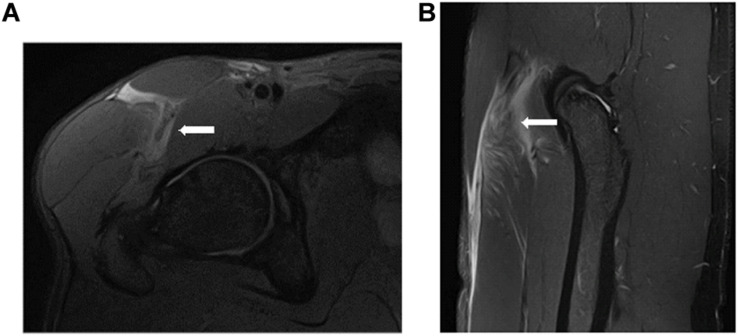
Proximal MTJ injury of rectus femoris [connective tissue tear (arrow), and muscle fibers damaged with loss of pennation angle] in axial **(A)** and sagittal **(B)** T2 weighted fat suppressed images.

**FIGURE 4:**
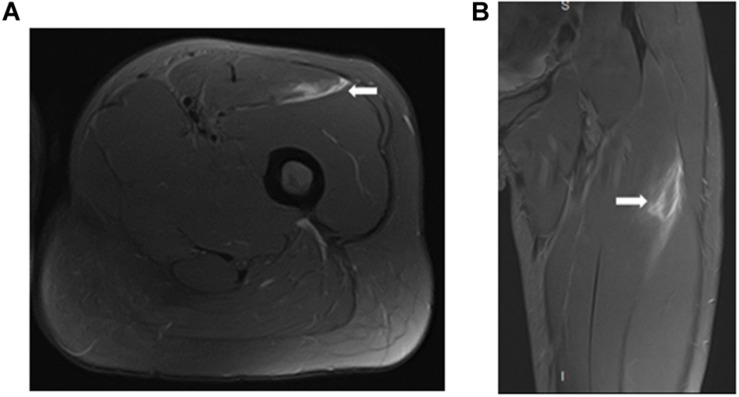
Distal MFJ injury of rectus femoris (arrow) in axial **(A)** and coronal **(B)** T2 weighted fat-suppressed image.

A total of 37 muscle injuries occurred between 2016–2017 and 2017–2018 seasons and evaluated by an MRI exam 24 h after injury, were grouped according to the myoconnective junction affected and classified following Pollock et al., 2014 in tendinous (*n* = 4), MTJ (*n* = 26), and MFJ (*n* = 7) injuries (Figures 2–4).

These injuries affected the long head of the biceps femoris (*n* = 12), the short head of the biceps femoris (*n* = 9), rectus femoris (*n* = 10), semitendinosus (*n* = 4) and semimembranosus (*n* = 2).

### L-BIA Measurements in Tendinous, MTJ and MFJ Injuries Diagnosed Previously by MRI 24 h After Injury

Table 1 shows the individual values of L-BIA measurements 24 h after injury [resistance (R), reactance (Xc), and phase angle (PA)] of thirty-seven muscle injuries. According to anatomical location, injuries are classified as tendinous (*n* = 4), MTJ (*n* = 26) and MFJ (*n* = 7) in). Grades of severity of MTJ injuries (MTJ-Grade 1, MTJ-Grade 2, MTJ-Grade 3) are also displayed.

**TABLE 1:** Individual values of L-BIA measurements 24 h after injury [contralateral non-injured side (1), injured side (2) in R, Xc, and PA], including tendinous (*n* = 4), MTJ (*n* = 26), and MFJ (*n* = 7) injuries.

**Muscle Injuries**		**R (Ω)**		**Xc (Ω)**		**PA** (°)	
	**Anatomical location**	**Non-injured side 24 h after injury (1)**	**Injured side 24 h after injury (2)**	**Non-injured side 24 h after injury (1)**	**Injured side 24 h after injury (2)**	**Non-injured side 24 h after injury (1)**	**Injured side 24 h after injury (2)**
1	T1	33.5	32.4	13.9	13.8	22.5	23.1
2	T2	38.8	38.0	17.2	17.0	23.9	24.1
3	T3	39.4	39.7	13.2	13.2	18.5	18.4
4	T4	45.5	44.4	12.8	12.8	15.7	16.1
5	MTJ-Grade 1	32.0	29.0	16.0	14.0	26.6	25.8
6	MTJ-Grade 1	29.0	26.0	14.0	12.0	25.8	24.8
7	MTJ-Grade 1	47.0	43.0	18.0	16.0	21.0	20.4
8	MTJ-Grade 1	31.0	28.0	15.0	13.0	25.8	24.9
9	MTJ-Grade 1	37.4	34.6	14.0	11.9	20.5	19.0
10	MTJ-Grade 1	48.7	41.5	15.0	12.6	17.1	16.9
11	MTJ-Grade 1	35.8	31.8	12.0	10.6	18.5	18.4
12	MTJ-Grade 1	42.0	40.1	18.9	17.5	24.2	23.6
13	MTJ-Grade 1	53.6	52.1	11.2	9.8	11.8	10.7
14	MTJ-Grade 1	39.1	37.1	15.7	14.5	21.9	21.3
15	MTJ-Grade 1	49.0	47.4	15.9	13.7	18.0	16.1
16	MTJ-Grade 2	42.0	37.0	17.0	13.0	22.0	19.4
17	MTJ-Grade 2	34.0	32.0	13.0	10.0	20.9	17.4
18	MTJ-Grade 2	38.6	35.0	15.2	12.0	21.5	18.9
19	MTJ-Grade 2	44.8	38.5	17.3	13.6	21.1	19.5
20	MTJ-Grade 2	59.4	52.0	14.2	11.2	13.4	12.2
21	MTJ-Grade 2	32.3	32.2	13.4	11.5	22.5	19.7
22	MTJ-Grade 2	41.0	38.0	13.2	11.0	17.8	16.1
23	MTJ-Grade 2	32.1	29.1	13.5	11.5	22.8	21.6
24	MTJ-Grade 3	29.3	24.6	15.3	9.7	27.5	21.6
25	MTJ-Grade 3	44.0	36.0	14.0	10.0	17.7	15.5
26	MTJ-Grade 3	39.6	27.7	14.3	7.3	19.8	14.6
27	MTJ-Grade 3	43.3	34.4	20.8	12.2	25.7	19.5
28	MTJ-Grade 3	42.9	40.3	12.9	9.8	16.7	13.7
29	MTJ-Grade 3	32.0	27.0	13.4	10.3	22.7	20.9
30	MTJ-Grade 3	43.2	37.6	16.7	12.3	21.1	18.1
31	MFJ	37.6	28.9	12.7	9.5	18.7	18.2
32	MFJ	68.0	54.0	19.0	13.0	15.6	13.5
33	MFJ	29.3	25.4	13.4	9.3	24.6	20.1
34	MFJ	37.2	32.4	15.0	11.1	22.0	18.9
35	MFJ	34.7	27.6	16.8	9.2	25.8	18.4
36	MFJ	47.3	40.4	18.5	12.7	21.4	17.5
37	MFJ	32.9	24.3	15.1	8.2	24.7	18.6

*R, Resistance; Xc, Reactance; PA, phase angle; T, tendinous; MTJ, myotendinous junction; MFJ, myofascial junction.*

Table 2 describes the L-BIA measurements 24 h after injury, of thirty-seven muscle injuries, as mean ± SD corresponding to tendinous (*n* = 4), MTJ (*n* = 26), and MFJ (*n* = 7) injuries and the percentage difference (Difference,%) in resistance (R), reactance (Xc), and phase angle (PA). In addition, the result of the repeated measures ANOVA test between injured side compared to the non-injured side of R, Xc, and PA is reported.

**TABLE 2:** Values of L-BIA measurements 24 h after injury [contralateral non-injured side (1), injured side (2) and % difference in R, Xc, and PA], and the result of repeated measures ANOVA test corresponding to tendinous, MTJ and MFJ.

**Tendinous, (*n* = 4)**	**Non-injured side 24 h after injury (1)**	**Injured side 24 h after injury (2)**	**Difference,%**	***F P value (1–2)^a1^***
R (Ω)	39.3 ± 4.9	38.6 ± 5.0	−1.8 ± 1.8	4.119 0.135
Xc (Ω)	14.3 ± 2.0	14.2 ± 1.9	−0.5 ± 0.6	2.455 0.215
PA (^0^)	20.2 ± 3.7	20.4 ± 3.8	1.2 ± 1.5	2.865 0.189

**MTJ, (*n* = 26)**	**Non-injured side 24 h after injury (1)**	**Injured side 24 h after injury (2)**	**Difference,%**	***F P value(1–2)^a2^***

R (Ω)	40.1 ± 7.7	35.8 ± 7.4	−10.7 ± 6.3	65.640 <0.001
Xc (Ω)	15.0 ± 2.2	12.0 ± 2.2	−20.0 ± 10.1	76.565 <0.001
PA (^0^)	20.9 ± 3.9	18.9 ± 3.8	−9.9 ± 7.0	41.988 <0.001

**MFJ, (*n* = 7)**	**Non-injured side 24 h after injury (1)**	**Injured side 24 h after injury (2)**	**Difference,%**	***F P value(1–2)^a3^***

R (Ω)	41.0 ± 13.1	33.3 ± 10.6	−18.7 ± 5.2	38.248 0.001
Xc (Ω)	15.8 ± 2.4	10.4 ± 1.9	−33.7 ± 8.4	73.600 <0.001
PA (^0^)	21.8 ± 3.7	17.9 ± 2.1	−17.0 ± 8.4	19.509 0.004

*^*a*^Repeated measures ANOVA test between non-injured side (1) and injured side (2) according to ^1^tendinous, ^2^MTJ, ^3^MFJ injuries. R, resistance; Xc, reactance; PA, phase angle; MTJ, myotendinous junction; MFJ, myofascial junction.*

Using repeated measures ANOVA test, anatomical changes related to tendinous injury were not reflected by L-BIA measurement through R, Xc, and PA (*P* > 0.05), 24 h after injury. The percentage difference in R, Xc, and PA, 24 h after injury, were −0.5% ∼−2%. However, we found statistical significance (*P* < 0.01) for R, Xc, and PA for both MTJ and MFJ injuries. The MTJ injury results showed statistical significance (*P* < 0.001) in percentage difference of R, Xc, and PA in the injured side compared to the contralateral non-injured side (−11% in R, −20% in Xc and −10% in PA), as well as MFJ injuries (*P* < 0.01) in percentage difference of R, Xc, and PA in injured side compared to the contralateral non-injured side (−19% in R, −34% in Xc, and −17% in PA).

The One-way ANOVA test, with regard to MTJ and MFJ injuries, shows statistical significance for% difference in R (*F* = 9.673, *P* = 0.004), Xc (*F* = 10.775, *P* = 0.003) and statistical significance for PA (*F* = 5.155, *P* = 0.030).

**FIGURE 5:**
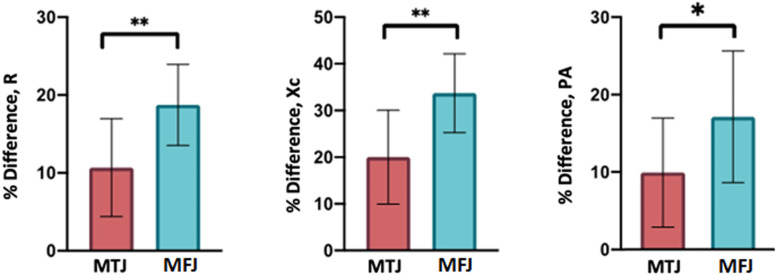
Mean and SD of the % difference in R, Xc, and PA of twenty-six MTJ (red) injuries and seven MFJ (blue). Results of One-way ANOVA between % difference of MTJ respect to MFJ injuries, are also presented.

Figure 5 shows the mean and SD of percentage difference (% Difference) in R, Xc, and PA, of MTJ and MFJ injuries, diagnosed by MRI exam 24 h after injury and evaluated by L-BIA measurements. In addition, the result of One-way ANOVA between % difference of R, Xc and PA in MTJ respect to % difference of R, Xc, and PA in MFJ injuries.

### L-BIA Measurements of MTJ Grouped According to the Severity of the Injury Diagnosed Previously by MRI 24 h After Injury

**FIGURE 6:**
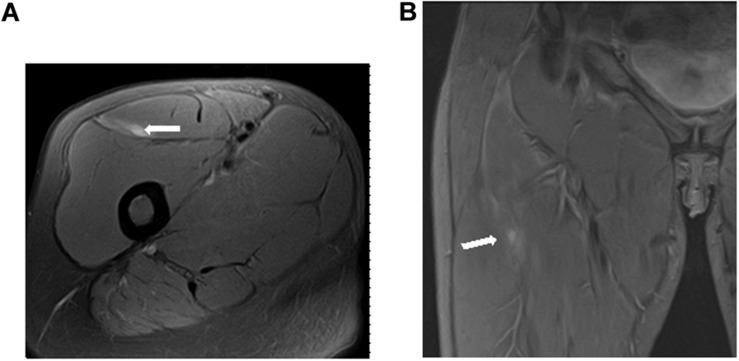
Distal MTJ injury grade 1 of rectus femoris in axial **(A)** and coronal **(B)** T2 weighted fat-suppressed image. Small myofascial tear (arrow).

**FIGURE 7:**
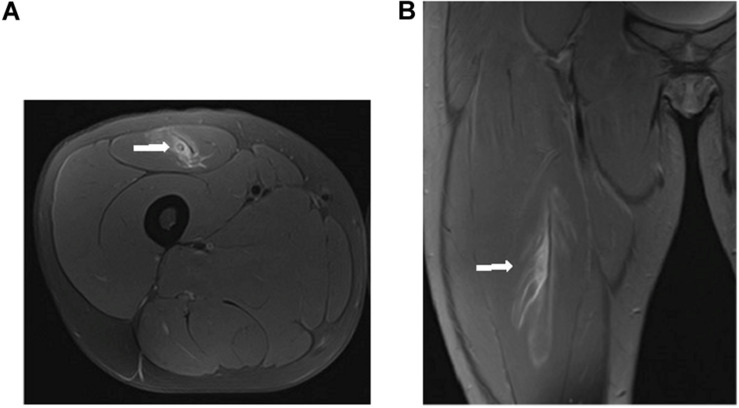
Proximal MTJ injury grade 2 of rectus femoris in axial **(A)** and coronal **(B)** T2 weighted fat-suppressed image. Blurring of the muscle fibers in the lateral MTJ of the central septum, with a small hematoma. We can see that this is an overloaded rectus femoris.

**FIGURE 8:**
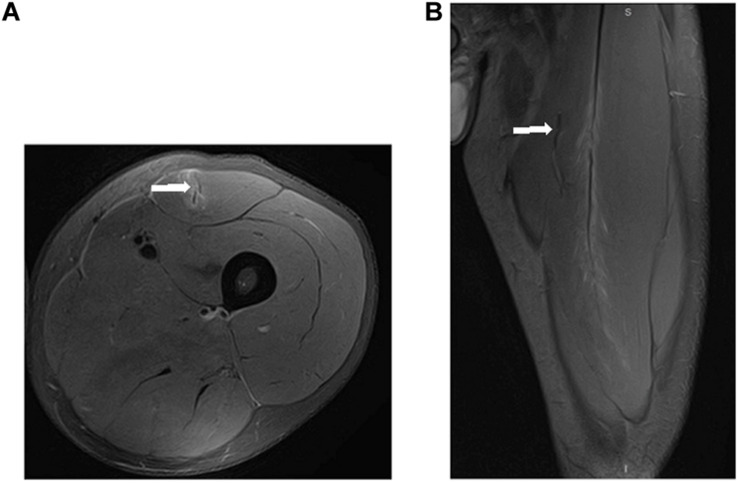
Proximal MTJ injury grade 3 of rectus femoris in axial **(A)** and coronal **(B)** T2 weighted fat-suppressed image. The mixed tear of the central septum (transversal and longitudinal), resulting in loss of tension and extensive feathery edema.

In a second analysis, 26 myotendinous junction (MTJ) injuries, evaluated by MRI 24 h after injury, were grouped according to the severity of the injury (Figures 6–8) follow BAMIC classification (Pollock et al., 2014) in grade 1 (*n* = 11), grade 2 (*n* = 8), and grade 3 (*n* = 7).

Table 3 presents the L-BIA measurements 24 h after the injury as mean ± SD and percentage difference (Difference,%) in R, Xc, and PA corresponding to the grade of severity of MTJ injury in grade 1 (*n* = 11), grade 2 (*n* = 8) and grade 3 (*n* = 7). In addition, the result of the repeated measures ANOVA test between injured side compared to the non-injured side of R, Xc, and PA is presented.

**TABLE 3:** Values of L-BIA measurements 24 h after injury [contralateral non-injured side (1), injured side (2) and % difference in R, Xc, and PA] corresponding to the grade of MTJ injuries, and the result of repeated measures ANOVA test for R, Xc, and PA.

**MTJ-Grade 1, (*n* = 11)**	**Non-injured side 24 h after injury (1)**	**Injured side 24 h after injury (2)**	**Difference,%**	***F P value(1–2)^b1^***
**R (Ω)**	40.4 ± 8.3	37.3 ± 8.4	−7.9 ± 3.7	40.405 <0.001
**Xc (Ω)**	15.1 ± 2.3	13.2 ± 2.3	−12.3 ± 2.8	229.221 <0.001
**PA (**°)	21.0 ± 4.5	20.2 ± 4.6	−4.3 ± 3.3	28.279 <0.001

**MTJ-Grade 2, (*n* = 8)**	**Non-injured side 24 h after injury (1)**	**Injured side 24 h after injury (2)**	**Difference,%**	***F P value(1–2)^b2^***

R (Ω)	40.5 ± 9.0	36.7 ± 7.0	−8.8 ± 4.4	20.788 0.004
Xc (Ω)	14.6 ± 1.7	11.7 ± 1.1	−19.5 ± 3.7	108.784 <0.001
PA (°)	20.3 ± 3.2	18.1 ± 2.9	−10.8 ± 3.5	54.428 <0.001

**MTJ-Grade 3, (*n* = 7)**	**Non-injured side 24 h after injury (1)**	**Injured side 24 h after injury (2)**	**Difference,%**	***F P value(1–2)^b3^***

R (Ω)	39.2 ± 6.0	32.5 ± 6.0	−17.1 ± 7.3	31.968 0.001
Xc (Ω)	15.3 ± 2.7	10.2 ± 1.7	−32.7 ± 9.8	42.505 0.001
PA (°)	21.6 ± 4.0	17.7 ± 3.1	−17.8 ± 6.6	32.618 0.001

*^*b*^Repeated measures ANOVA test between non-injured side (1) and injured side (2) according to grade of MTJ injuries ^1^grade 1, ^2^grade 2, ^3^grade 3. R, resistance; Xc, reactance; PA, phase angle; MTJ, myotendinous junction; MFJ, myofascial junction.*

Using repeated measures ANOVA test, anatomical changes related to the grade of MTJ injuries were reflected by L-BIA measurement through R, Xc, and PA (*P* < 0.01), 24 h after injury for grade 1, 2, and 3.

The One-way ANOVA test, regarding the MTJ injuries classified in grades 1, 2, and grade 3, showed statistical significance in the % difference of R (*F* = 7.630, *P* = 0.003) and statistical significance for % difference of Xc (*F* = 26.964, *P* < 0.001) and % difference of PA (*F* = 20.089, *P* < 0.001).

**FIGURE 9:**
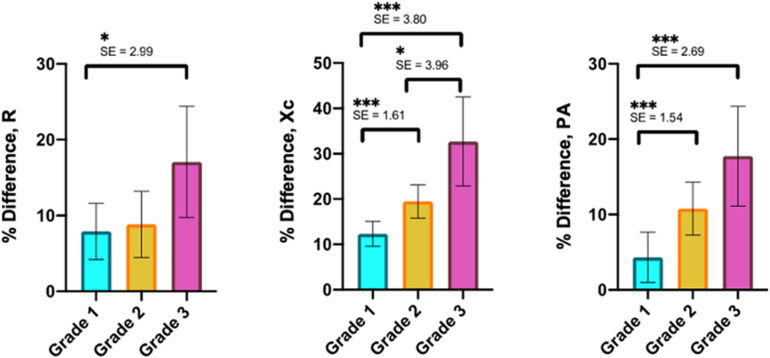
Mean and SD of % difference in R, Xc, and PA of MTJ injury grades 1–3. SE and results from *post hoc* test are also presented.

Figure 9 shows the mean and SD of the % difference in R, Xc, and PA, of twenty-six MTJ injuries classified as grade 1 (*n* = 11), grade 2 (*n* = 8), and grade 3 (*n* = 7) by MRI exam, 24-hour after injury. In addition, the results of Tamhane’s T2 multiple comparison test revealed statistical differences (*P* < 0.001) in Xc and PA between grade 1 and grade 2, as well as between grade 1 and grade 3.

**FIGURE 10:**
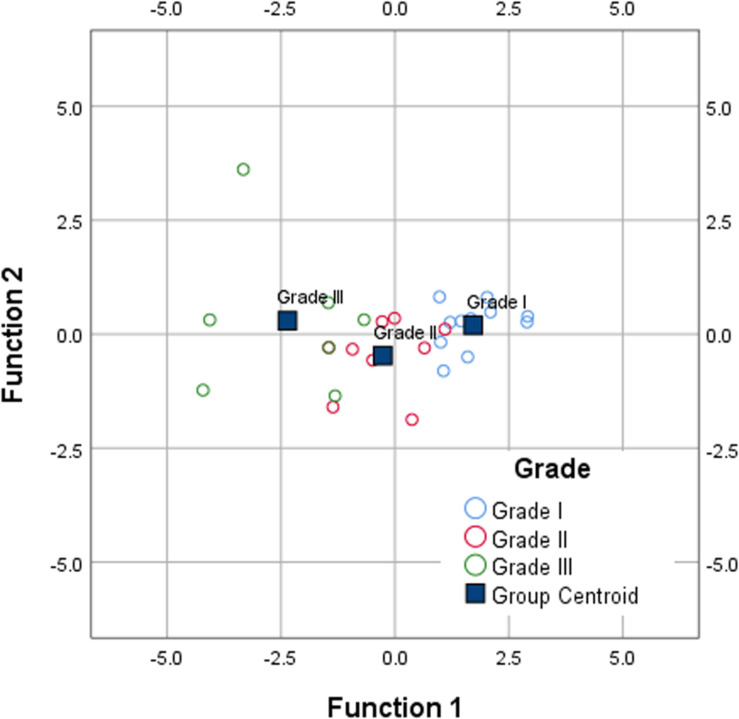
Canonical discriminant functions for % difference in R, Xc, and PA, according to the grade of MTJ injuries, of grade 1 (*n* = 11), grade 2 (*n* = 8), and grade 3 (*n* = 7).

Figure 10 shows the canonical discriminant functions, according to the grade of MTJ injuries, by Discriminant Function Analysis of percentage difference (% difference) in R, Xc, and PA.

The function scores obtained through the standardized canonical discriminant function coefficients are:

F⁢S⁢c⁢o⁢r⁢e⁢1=3.198%⁢difference⁢R-5.093%⁢difference⁢Xc

+2.701%⁢difference⁢PA

F⁢S⁢c⁢o⁢r⁢e⁢2=4.212%⁢difference⁢R-4.746%⁢difference⁢Xc

+3.064%⁢difference⁢PA

Both first and second function show the standardized coefficient for % difference R, % difference Xc, and % difference PA. The magnitude of these coefficients indicate how strongly the discriminating variables affect the score. Thus, % difference in Xc has the greatest impact of the three variables, on the first (−5.093) and second (−4.746) discriminant score, but especially in the first score. Therefore, Xc is the variable that most discriminates according to the grade of MTJ injuries by L-BIA measurements 24 h after injury.

### Return to Play (RTP) in MTJ and MFJ Injuries

Table 4 present the RTP (in days), and the result of the One-way ANOVA test between MTJ and MFJ injuries and according to the grade of severity of MTJ injuries.

**TABLE 4:** Return to play in MTJ and MFJ injuries.

**Anatomical Location (Sample size)**	**RTP, days**	***F P value*^1^**
MTJ (26)	25 ± 22	2.277 0.124
MFJ (7)	20 ± 8	

**Grade of MTJ injury (Sample size)**	**RTP, days**	***F P value*^2^**

Grade 1 (11)	8 ± 3	39.517 *P* < 0.001
Grade 2 (8)	14 ± 10	
Grade 3 (7)	52 ± 14	

*^1^One-way ANOVA between MTJ and MFJ injuries. ^2^One-way ANOVA among MTJ injuries according to severity.*

Using the One-way ANOVA test, and regarding MTJ and MFJ injuries, the RTP does not show statistical significance (*P* > 0.05).

Regarding the MTJ injuries classified in grades 1, 2, and grade 3, we found statistical significance in days to RTP (*P* < 0.001). Tamhane’s T2 reveals statistical difference (*SE* = 5.121, *P* = 0.002) contrasting grade 1 (8 ± 3 days) with grade 3 (52 ± 14 days) and also statistical difference (*SE* = 5.475, *P* = 0.001) contrasting grade 2 (14 ± 10 days) with grade 3 (52 ± 14 days).

## Discussion

This second project of L-BIA developed by FCB Medical Department evaluates the capacity of the L-BIA method to differentiate muscle injuries according to anatomical location in tendinous, MTJ and MFJ, which are diagnosed by MRI exam 24 h after injury and classified according to *The British Athletics Muscle Injury Classification* (Pollock et al., 2014) considering the histoarchitectural approach to skeletal muscle injury (Balius et al., 2020). The most important finding is the good association between both MRI and L-BIA methods in myotendinous junction (MTJ) and myofascial junction (MFJ) injuries. Also, the fact that higher changes in Xc, and longer time to RTP are found in more severe MTJ injuries.

Comparing the injured side to non-injured side both for MTJ and MFJ injuries, the % difference in L-BIA measurements 24 h after injuries are in concordance with findings by MRI exam 24 h after injury. The contralateral non-injured side is taken as reference due to the individual symmetry in L-BIA values between left and right muscle areas of the lower-limb in professional football players. By MRI exam, MFJ injuries show greater edema and greater bleeding than MTJ injuries, which justifies why the percentage difference (% difference) of the injured side compared to the contralateral non-injured side is greater in MFJ injuries than in MTJ injuries. Both in MTJ and MFJ injuries the % difference in Xc was greater than the % difference in R. According to our findings, to evaluate anatomical changes produced in MTJ and MFJ, 24 h after injury by L-BIA, the Xc is the most sensitive variable.

Although the tendinous injuries are the most severe cases (Crema et al., 2016) L-BIA method is completely insensitive to the anatomical changes produced in the tendinous injuries. Fortunately, they are also less common in professional football players (Crema et al., 2016). By MRI, the tendinous injuries show less edema and bleeding comparing to MTJ and MFJ injuries, and the intra-tendinous gap is considerably smaller. A normal tendon is a compact structure with tenocytes attached to a highly ordered fibrillar collagen matrix (ECM) composed of type-I collagen (65–80% of its dry mass), and small leucine-rich proteoglycans that regulate collagen self-assembly into collagen fibrils, which in turn are ordered by the cell into collagen fibers (Snedeker and Foolen, 2017). In the intratendinous injury, tendon gap is smaller than those in muscle injuries and show generally less edema and bleeding than MTJ and MFJ injuries in MRI fluid-sensitive sequences. In acute intratendinous injuries, there is no major inflammatory component and its repair process goes along the lines of scar formation causing to the tensile strength of healed tendon like the healthy tendon (Nourissat et al., 2015). It should be clarified that differently to the hamstring free tendon, the intramuscular tendon has more vascularization (Brukner and Connell, 2016).

Comparing the percentage difference of MTJ injuries to the percentage difference of MFJ injuries, as in MRI exam 24 h after injury, both for edema produced in the area of the injury (% difference R) and the amount of cell disruption (% difference Xc), the L-BIA method distinguishes between MTJ and MFJ injuries. The % difference in phase angle is more closely related to the changes produced in the Xc than with those produced in R for the geometric relationship between them (Lukaski, 1996; Lukaski et al., 2019).

In professional football, muscle injuries occur with major frequency at the myotendinous junction (MTJ) especially in hamstring muscle groups (Crema et al., 2016) whose main objective is the transmission of force (Balius et al., 2018). In this study, 26 out of 37 injuries occurred in the myotendinous junction, which were classified into grades 1, 2, and 3, according to the severity of the injury. All of them showed a notable decrease in R and Xc, being the largest change observed in Xc. Furthermore, this change was even greater in grade 3 of the MTJ injury after comparing the injured side to the non-injured side. The % difference of MTJ injuries grade 1 compared to the % difference of MTJ injuries grade 2 and MTJ grade 3, elucidated that the more severe the injury is, higher changes in R and Xc are found, especially in Xc which is relate to muscle “gap” in agreement with the findings shown on the MRI exam 24 h after injury. Similar to wound healing (Lukaski and Moore, 2012), muscle injury is classified according to anatomical location, the % difference in Xc (cell disruption, muscle “gap”) is related with the severity of the injury. Regarding RTP (in days), it was not found differences between MTJ and MFJ injuries. However, according to severity of MTJ injuries, it was found differences among days to RTP and MTJ grades 1, 2, and 3. The injuries with the highest degree of severity were associated with longer time to RTP.

The decision on the days to RTP, is conditioned by the severity of the injury (Pedret et al., 2015; Balius et al., 2018, Balius et al., 2020) and by the myoconnective junction involved. The days to RTP are strongly associated with changes in Xc (muscular fibers retraction quantified by % different of Xc) i.e., the severity of injuries is reflected by higher changes in Xc and longer time to RTP. Injury recovery is related to a readjustment of the Xc parameter closer to the value of the contralateral non-injured side with % difference lower than 15% in those muscle injuries of high degree of severity according to the muscle “gap” (Nescolarde et al., 2013). It is important mentioning that for measurements on injured muscles with L-BIA until RTP time point, each player is his own reference (Nescolarde et al., 2013). The role of MRI is key for the diagnosis and prognosis of injuries, and therefore, a great tool for RTP prediction. However, it is not the only criterion to take into account. The decision on time to RTP by FCB physicians are based on club’s guidance, taking into account not only imaging criteria but also specific tests, GPS assessment, and even the player’s psycho-emotional state.

## Contribution

Localized bioimpedance is a complementary method to MRI and US as currently it can only quantify MTJ and MFJ injuries. In addition, it could be useful for the medical team, physiotherapists, and physical trainers in the diagnosis of muscle injury helping in day-to-day decision-making. L-BIA measurements are carried out using a phase-sensitive impedance instrument at 50 kHz, robust and easy to apply, and economically feasible.

## Limitation

To validate L-BIA as a complementary method to MRI and US, it is necessary to extend this study to other professional football teams to increase the sample size, as well as to include female athletes.

## Conclusion

We conclude that L-BIA is a tool able to differentiate between MTJ and MFJ injuries. Additionally, it discriminates between MTJ grade 3 injury and grades 1 and 2. The sensitivity of L-BIA method is shown to be higher in the percentage difference of Xc 24 h after injury, which is related to muscle cell disruption. In addition, more severe injures presented higher % difference in Xc and longer time to RTP.

## Data Availability Statement

The raw data supporting the conclusions of this article will be made available by the authors, without undue reservation.

## Ethics Statement

Ethics approval was obtained from the Medical Committee of Barça Innovation Hub in Futbol Club Barcelona according to principles of the Helsinki Declaration for experiments with human beings. The participants provided oral and written informed consent before participation in the study during a period of 2016–2017 and 2017–2018. The patients/participants provided their written informed consent to participate in this study.

## Author Contributions

All authors designed the experiments, analyzed the data, revised the manuscript, and approved the final version of the manuscript. LN processed the data and prepared the tables and figures. LN and GR wrote the manuscript.

## Conflict of Interest

The authors declare that the research was conducted in the absence of any commercial or financial relationships that could be construed as a potential conflict of interest.
